# Interleukin 12 a Key Immunoregulatory Cytokine in Infection Applications

**DOI:** 10.3390/ijms11030789

**Published:** 2010-02-26

**Authors:** Therwa Hamza, John B. Barnett, Bingyun Li

**Affiliations:** 1 Biomaterials, Bioengineering & Nanotechnology Laboratory, Department of Orthopaedics, School of Medicine, West Virginia University, Morgantown, WV 26506, USA; E-Mail: thamza@hsc.wvu.edu (T.H.); 2 Pharmaceutical and Pharmacological Sciences Graduate Program, Health Sciences Center, West Virginia University, Morgantown, WV 26506, USA; 3 Department of Microbiology, Immunology, and Cell Biology, West Virginia University, Morgantown, WV 26506, USA; E-Mail: jbarnett@hsc.wvu.edu (J.B.B.); 4 WVNano Initiative, Morgantown, WV 26506, USA; 5 Department of Chemical Engineering, College of Engineering and Mineral Resources, West Virginia University, Morgantown, WV 26506, USA

**Keywords:** interleukin 12, infection, interferon-γ, cell-mediated immunity, cell signaling

## Abstract

Interleukin 12 (termed IL-12p70 and commonly designated IL-12) is an important immunoregulatory cytokine that is produced mainly by antigen-presenting cells. The expression of IL-12 during infection regulates innate responses and determines the type of adaptive immune responses. IL-12 induces interferon-γ (IFN-γ) production and triggers CD4^+^ T cells to differentiate into type 1 T helper (Th1) cells. Studies have suggested that IL-12 could play a vital role in treating many diseases, such as viral and bacterial infections and cancers. The unique heterodimeric structure, which IL-12 shares with its family members including IL-23, IL-27, and IL-35, has recently brought more attention to understanding the mechanisms that regulate the functions of IL-12. This article describes the structure and biological activities of IL-12 in both the innate and adaptive arms of the immune system, and discusses the applications of IL-12 in treating and preventing infections.

## 1. Introduction

It is well-established that CD4^+^ T helper (Th) cells can be divided, besides T regulatory and Th17, into two major subsets: Th1 and Th2, based on their patterns of cytokine production. Th1 cells mainly secrete interleukin 2 (IL-2), IFN-γ, and IL-12, whereas Th2 cells secrete IL-4, IL-5, and IL-10 [1]. Functionally, Th1 cells predominantly promote cell-mediated immunity and help in clearance of intracellular pathogens; Th2 cells are responsible for humoral immunity protecting against extracellular invaders. The balance between IL-12, favoring Th1 responses, and IL-4, favoring Th2 responses, determines the early preference expressed in the immune response.

IL-12 has multiple biological functions and importantly, it bridges the early nonspecific innate resistance and the subsequent antigen-specific adaptive immunity [2]. IL-12 was first identified as a product of Epstein-Barr virus (EBV)-transformed human B cell lines. IL-12 was previously known as T cell differentiation factor (TCDF) or natural killer cell stimulatory factor (NKSF). IL-12 used to be the only known heterodimeric cytokine, but it is now a part of a family consisting of several other members including IL-23, IL-27, and the recently identified IL-35. These new molecules have been found to play distinct cellular and functional roles in Th1 development [3]. In this article, the structure and functions of IL-12 are reviewed and the role of IL-12 in regulating immune responses and treating infections is discussed.

## 2. IL-12 Molecular Structure and Signaling Pathway

### 2.1. IL-12 Molecular Structure

**Figure 1.**
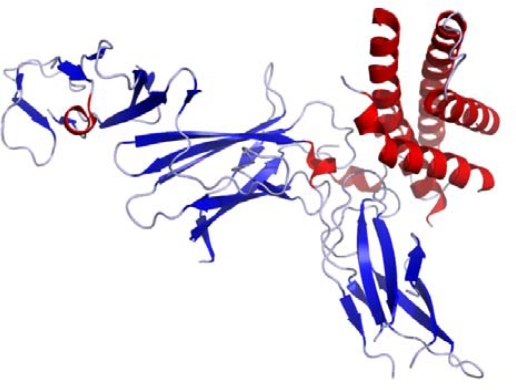
Crystal structure of IL-12. IL-12 is composed of a bundle of four alpha helices. It is a heterodimeric cytokine encoded by two separate genes, IL-12p35 and IL-12p40. Obtained from the Protein Data Bank (PDB: 1F45).

IL-12 is a key immunoregulatory cytokine [2,4,5] with a molecular weight of 70 kDa (Figure 1), composed of two covalently-linked subunits, IL-12p35 (35 kDa) and IL-12p40 (40 kDa), each of which is expressed on different chromosomes. The sequence of the p35 gene is homologous to that of IL-6 and granulocyte-colony stimulating factor [6]. The sequence of the p40 chain has a homology to the extracellular domain of the IL-6 receptor (IL-6R) α-chain and the ciliary neurotropic factor [7]. This explains some of the redundant actions of these cytokines.

Although p35 transcripts are found in many cell types, free p35 is not secreted without the p40 subunit. IL-12p40 is produced predominantly by activated monocytes, macrophages (MΦs), neutrophils, and dendritic cells (DCs). It has been suggested that in mice, but not in humans, IL-12p40 homodimers antagonize IL-12p70 activity by binding to the β1 subunit of the IL-12 receptor [8–10]. Also, IL-12p40 has been shown to act as a chemoattractant for MΦs and promotes the migration of stimulated dendritic cells [11]. The p40 subunit is associated with several pathogenic inflammatory responses such as silicosis, graft rejection and asthma, but it is also found to be protective in a mycobacterial infection model [11].

The biological activities of IL-12 are mediated *via* binding to a membrane receptor complex which is also composed of two subunits: IL-12R β1 and IL-12R β2. Both of the subunits are members of the class I cytokine receptor family, which includes IL-6, IL-11, and leukocyte inhibitory factor related to glycoprotein gp130 [12,13]. There is a high level of conservation, with a 68% amino acid sequence homology, between mouse and human IL-12R β2 proteins, and a 54% sequence identity homology between the mouse and human IL-12R β1 [12]. Both receptor chains are required to mediate maximal signaling; however, the two chains have independent roles. IL-12R β1 is required for high-affinity binding to the IL-12p40 subunit and it is associated with the Janus kinase (Jak) family member Tyk-2, while the IL-12R β2 chain mediates signal transduction *via* three tyrosine residues that act as a docking site for STAT4 and is associated with Jak-2. IL-12R β2 recognizes either the heterodimer IL-12 or the IL-12p35 subunit and is expressed, not on naive T cells, at low levels after T cell receptor stimulation. Expression of this receptor subunit is critically influenced by IL-12 and type 1 IFNs [5]. The initial expression of functional IL-12Rs is further enhanced when IL-12 is present at the time of priming, working as a positive feedback loop regulator [14].

### 2.2. IL-12 Family Members

**Figure 2.**
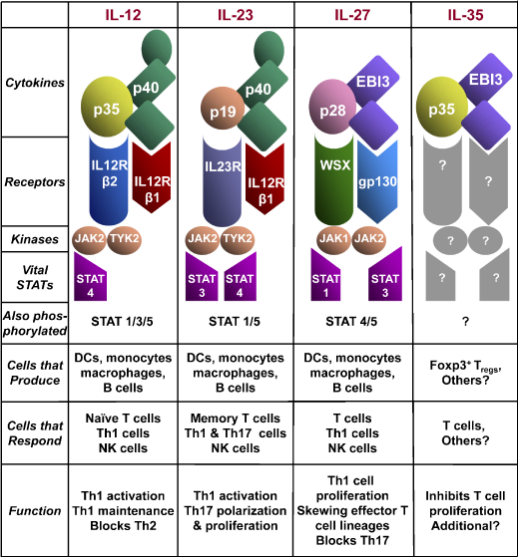
The IL-12 cytokine family; structural and biological characteristics. Reprinted from Ref. [13].

The IL-12 family has expanded recently to include members like IL-23, IL-27, and IL-35. IL-12 and these new family members play critical roles in Th1 cell development [4,15]. Figure 2 summarizes the structure and biological properties of these major IL-12 family members.

#### 2.2.1. Interleukin-23

IL-23 is a proinflammatory cytokine that is closely related in structure to IL-12; they both share the p40 subunit that is able to build a disulfide bridge complex with the p19 subunit to form the biologically active molecule. The p40-p19 complex is mainly secreted by activated intestinal antigen presenting cells, such as DCs and monocytes [15]. Similar to IL-12 signaling, IL-12R β1 is required for high affinity binding to p40, whereas IL-23R has a cytoplasmic STAT4 binding domain for signal transduction. However, STAT3/STAT4 heterodimers in IL-23 instead of STAT4 homodimers in IL-12 are translocated to the nucleus to induce specific gene expression [16].

The main role of IL-23 involves the stimulation of Th17 cells to produce IL-17 [3]; it also induces the proliferation of memory T cells [17]. Unlike IL-12, IL-23 does not induce significant production of IFN-γ and in the absence of IL-23, IFN-γ production and Th1 differentiation are still found to be normal [15].

#### 2.2.2. Interleukin-27

IL-27 is another heterodimeric cytokine composed of the p28 subunit and the p40-related protein Epstein-Barr virus-induced gene 3 (EBi3). IL-27 activities are driven *via* one receptor that was described recently with some homology to gp130, known as TCCR or WSX-1 [18]. Signaling through TCCR or WSX-1 is required for the early initiation of a Th1 response, but is not necessary for the maintenance of Th1 responses [18]. The signaling pathway initiated by IL-27 activates Jak-1 and Jak-2 molecules. However, only STAT1 and STAT3 are critical to IL-27 bioactivity, and STAT3 rather than STAT1 is required for IL-27-induced proliferation [19].

IL-27 is rapidly produced by human phagocytic cells and DCs after their activation [18]. IL-27 is involved in early Th1 initiation, is able to induce proliferation of naive but not memory T cells, and can synergize with IL-12 in IFN-γ production [19].

#### 2.2.3. Interleukin-35

IL-35 was discovered very recently [20]. This heterodimeric cytokine is composed of the p35 subunit and the p40-related protein EBi3. The IL-35 R complex has not been characterized yet, but it is possible that it may be composed of the receptors known for IL-27 and IL-12, either gp130 or WSX-1 and IL-12R β2.

Within the CD4^+^ T-cell population, IL-35 is expressed by resting and activated T regulatory cells (Tregs) but not activated T cells. Loss of IL-35 expression results in reduced immune suppression by Tregs. IL-35 appears to function as an anti-inflammatory molecule by inhibiting T-cell proliferation. It is suggested that IL-35 can suppress Th17 development *in vivo* [21]; however, further studies are required to fully understand the exact mechanism behind these functions.

### 2.3. IL-12 Signaling Pathway

**Figure 3.**
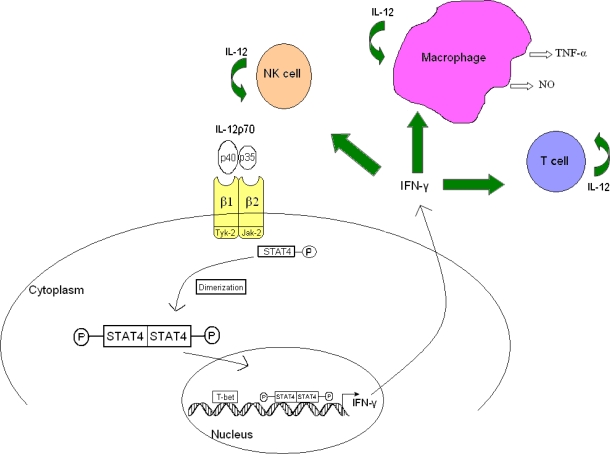
IL-12 signaling pathway. IL-12 activates the Jak/STAT pathway. Following binding of IL-12p40 and IL-12p35 to IL-12R β1 and IL-12R β2, respectively, Jak-2 and Tyk-2 get transphosphorylated. Phosphorylated IL-12R β2 binds to STAT4 which will then dimerize with another STAT4 molecule. STAT4 homodimers translocate to the nucleus and promote IFN-γ gene transcription. T-bet is another transcription factor that plays a role in Th1 development and IFN-γ production. The IL-12 and IFN-γ induce the activity and proliferation of MΦs, NK cells, and T cells, which also secrete IL-12. Modified from Ref. [34].

The signaling pathway of IL-12 is described in Figure 3. On binding of IL-12 to the IL-12R complex, activation of Jak kinases (Tyk-2 and Jak-2) occurs, leading to phosphorylation of the receptor, which becomes a binding site for STAT4 proteins that are rapidly recruited to the receptor and phosphorylated on their tyrosine residues by the Jak kinases. Tyrosine phosphorylation of STAT4 proteins induces their homodimerization and translocation to the nucleus where they bind to specific sequences and regulate gene transcription (Figure 3). In Th1 and NK cells, IL-12 mainly induces STAT4 activation. In addition, STAT1, STAT3, and STAT5 can also be activated by IL-12 signaling [13,22]. Studies have shown that IFN-γ increases the transcription factor T-bet activity, which was originally shown to be induced in Th1 cells [23]. T-bet response to IFN-γ leads to the up-regulation of IL-12R β2 surface expression and allows for Th1 cell responsiveness to IL-12 [23,24]. More recent studies showed that T-bet negatively regulates GATA-3 expression, the main regulator of Th2 cell axis [25]. In contrast, the Th2 cytokine IL-4 reduces IL-12R β2 expression and thus leaves Th2 cells non-responsive [26]. The opposite effects of IFN-γ and IL-4 on IL-12R β2 expression may contribute to the commitment of Th1/Th2 differentiation. Priming of macrophages with IFN-γ increases cellular responsiveness to inflammatory stimuli, including IFN-γ itself, which activates direct microbicidal functions including the production of reactive oxygen species and TNF-α and promotes the antigen (Ag) processing and presentation capacities of macrophages. Also, IFN-γ plays a role in the regulation of T cell proliferation and activity [27].

Recently, Toll like receptors (TLRs) are believed to play an important role in the balance between production of IL-12 and its family members (e.g., IL-23 and IL-27) [28]. TLRs are part of the innate immune system that recognizes pathogen antigens causing a proper immune response activation. The binding of pathogen-associated molecular patterns to different TLRs can stimulate the production of IL-12 family members. However, engagement of two or more TLRs can induce more significant levels of IL-12 than activation of a single TLR; coupling of TLR8 ligands with TLR3 or TLR4 induces higher levels of IL-12 than stimulation by individual TLR ligands [29]. Importantly, selective production of each of IL-12 family member is regulated by triggering specific TLRs. For instance, the activation of TLR4 can induce the production of both IL-12 and IL-23, whereas activation of TLR2 induces high levels of IL-23 but not IL-12 [30]. On transcription regulation level, signaling through TLRs leads to different gene-inducing programs. TLRs (except TLR3) bind to the adapter molecule MyD88 and result in nuclear factor-κB (NF-κB) activation. This signaling pathway has been shown to activate the genes encoding the subunits of IL-12, IL-23, and IL-27 [31–33]. Several other transcription factors, such as IFN-regulatory factors (IRFs) are involved in IL-12 production, including IRF1, IRF3, and IRF7. Also, IFN-γ signaling through IRF1 and IRF8 triggers TLR-induced IL-12 production [28].

## 3. IL-12 Biological Activities

**Figure 4.**
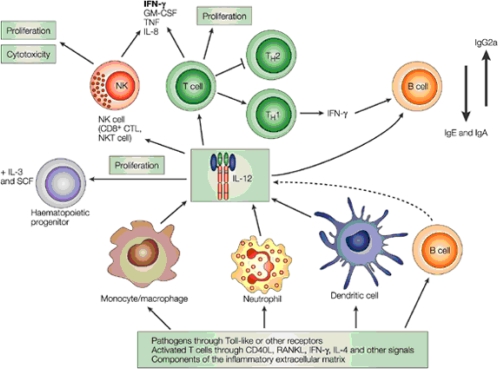
Summary of the biology of IL-12 [14]. The main physiological producers of IL-12 are phagocytes (monocytes/MΦ and neutrophils) and DCs in response to pathogens (bacteria, fungi, intracellular parasites and viruses) through TLRs and other receptors, to membrane-bound and soluble signals from activated T cells and NK cells, and to components of the inflammatory extracellular matrix (for example, low-molecular-weight hyaluronan) through CD44 and TLRs. The physiologically most important target cells of IL-12 are: haematopoietic progenitors, for which, in synergy with other colony-stimulating factors, IL-12 induces increased proliferation and colony formation; NK cells, NK T cells and T cells, for which IL-12 induces proliferation, enhancement of cytotoxicity and of the expression of cytotoxic mediators, and the production of cytokines, particularly IFN-γ, as well as favoring differentiation to cells that produce type-1 cytokines (Th1, TC1 and NK1 cells); and B cells, for which IL-12, directly or through the effects of type-1 cytokines such as IFN-γ, enhances the activation and production of Th1-associated classes of immunoglobulin (for example, IgG2a in the mouse). CTL, cytotoxic T lymphocyte; GM-CSF, granulocyte macrophage colony-stimulating factor; RANKL, receptor activator of nuclear factor-κB ligand; SCF, stem-cell factor; TC1, T cytotoxic 1; TH1/Th1, T helper 1; TNF, tumor-necrosis factor. Reprinted from Ref. [14].

IL-12 is produced mainly by DCs, MΦs, monocytes, neutrophils, microglia cells and, to a lesser extent, by B cells (Figure 4); human but not murine B cells were found to produce IL-12 following CD40 ligation [35]. Non-immune cells such as infected-keratinocytes and osteoblasts, epithelial and endothelial cells have also been shown to produce some amounts of this cytokine [36,37]. Pathogen-associated molecular patterns such as lipopolysaccharide (LPS), teichoic acid, peptidoglycan, and bacterial CpG DNA, can induce IL-12 production. The production of IL-12 is regulated by positive and negative feedback mechanisms involving Th1 cytokines (e.g., IFN-γ), Th2 cytokines (e.g., IL-10), and type 1 IFN (Figure 4) [38,39].

IL-12 has multiple biological functions (Table 1) and it bridges innate and adaptive immunity. IL-12 induces differentiation of naive CD4^+^ T cells to Th1 cells and activates NK cells. Upon activation, these cells produce IFN-γ and other type-1 cytokines [40]. IL-12 also protects CD4^+^ Th1 cells from antigen-induced apoptotic death [41]. IL-12 was found to have synergistic effects with IL-18 in developing Th1 cells, and IL-12 and IL-18 reciprocally upregulate each other’s receptors [5]. In addition, IL-12 plays a role in T cell trafficking and migration by inducing functional adhesion molecules such as P- and E-selectin ligand expression on Th1 cells but not Th2 cells; therefore, these cells are selectively recruited to sites where Th1 immune responses are needed [42]. Studies also reported selective expression of CCR5 and CXCR3 when naive T cells were primed in the presence of IL-12 [40,43]. Stimulation of MΦ-derived IL-12 also plays a major role in the induction of resistance in parasitic infestation [44].

The biological activity and quantity of IL-12 can be determined using molecular approaches. One of the first approaches for quantifying IL-12 was based on IL-12-induced proliferation of phytoheagglutinin (PHA)-stimulated lymphocytes [45]. Peripheral blood mononuclear cells activated with PHA along with IL-2 proliferate in response to IL-12. Enzyme-linked immunosorbent assay (ELISA) was also utilized to quantify specifically IL-12p40 and IL-12p70 [46]. In combination with ELISA, another approach, based on the ability of IL-12 to induce IFN-γ secretion from activated T-lymphoblasts, was developed and has been used to measure the biological activities of IL-12 [47,48].

## 4. IL-12 Therapeutic Applications in Infections

IL-12 holds considerable promise as an immunotherapeutic agent because it plays a central role in regulating innate and adaptive immune responses, and synergizes with several other cytokines for increased immunoregulatory activities. Also, tipping the balance between the expression of IL-12 family members may represent a novel strategy to control T-cell-mediated immune responses [28]. Animal and human studies have shown improved outcomes in treating or preventing infections based on the mechanisms of IL-12-dependent therapies.

### 4.1. Viral Infections

The role of IL-12 in promoting endogenous protective immune responses to viral infections has been attracting more attention with time. Many experimental models have been developed to test the potential effects of IL-12 and other immune mediators on the herpes simplex virus, influenza virus, human immunodeficiency virus (HIV), lymphocytic choriomeningitis virus, mouse cytomegalovirus, and many others [49].

#### 4.1.1. Herpes simplex virus

In an *in vivo* herpes simplex virus-1 (HSV-1) mouse model, sustained expression of IL-12 was induced by the virus. This local IL-12 production was considered to cause an immunopathological response in the mouse eye environment due to Th1 immunity [50]. However, exogenous IL-12 showed a protective role and increased the resistance to infection in HSV-1-thermally injured mice because of the activation of viral-specific Th1 immunity [51]. Also, IL-12 and IL-18 were shown to play an important role in innate immunity against genital HSV-2 infection in a mouse model; the protection was mainly mediated by IFN-γ production [52].

#### 4.1.2. Influenza virus

CD4^+^ T cells are important in controlling influenza A virus infection. CD4^+^ T cells drive the induction and expansion of CTL against such viral pathogens. Earlier studies using the influenza virus system as a model showed that DCs induced effective CTL response without increasing endogenous IL-12 levels. Exogenous application of IL-12 was found to significantly enhance CTL activity [53]. Studies also demonstrated a combined effective role of IL-12 and DCs in enhancing CTL immunity to viral infection [53]. Influenza virus vaccination effectively protects individuals against serious complications through induction of humoral and cellular responses [54]. Interestingly, patients identified with genetic IL-12/23R β1 or IFN-γR deficiencies were found to develop immune responses to childhood vaccination [53]. Also, the influenza vaccination could induce humoral immunity and IFN-γ production in IL-12/23R β1 deficient patients [55]. These studies may imply that both IL-12 and IFN-γ play a significant role in fighting viral infections.

#### 4.1.3. Human immunodeficiency virus (HIV)

HIV infection mainly induces Th2 immunity which, most of the time, is unable to clear the viral infection. Glycoprotein gp120 of HIV has been shown to induce IFN-γ dependent production of IL-12 in cultured MΦs. The use of IL-12 production and Th1 activation might contribute to decreased viral load and increased clearance of the infection [56]. However, phase I trials in HIV patients have shown moderate effects accompanied by some toxic responses [57].

### 4.2. Bacterial Infections

As a potent inducer of Th1 immune response and an important mediator between innate and adaptive immunity, IL-12 has potential clinical uses in treating and preventing bacterial infections.

#### 4.2.1. Bone infection

Osteomyelitis is a bone infection characterized by the presence of necrotic bone tissue and increased osteoclast activity. It is commonly caused by *Staphylococcus aureus* (*S. aureus*), which is traditionally known as an extracellular pathogen; however, it has been shown that *S. aureus* can be internalized and live within a variety of host cells [58,59]. Internalization of *S. aureus* into the host cells could be an important pathogenicity factor for escaping the host immune system leading to persistence of infection [60]. It was reported that mouse and human osteoblasts *in vitro* infected with *S. aureus* express high levels of IL-12 [37], and IL-12 and/or IL-23 knockout mice showed an increased susceptibility to bacterial, parasitic, and fungal infections [61]. The ability of *S. aureus*-infected osteoblasts to elaborate bioactive IL-12, which may augment a Th1 immune response, has important implications for host defense against intracellular pathogens such as the internalized *S. aureus*, and such a mechanism would seem to be a significant advantage for the elimination of intracellular bacteria [60]. IL-12 may also play a critical role in the downregulation of *S. aureus* growth *in vivo* [62]. Of note, the role of IL-12 and Th1 cells in the development of cell-mediated inflammatory disorders is still not clear [28].

**Figure 5.**
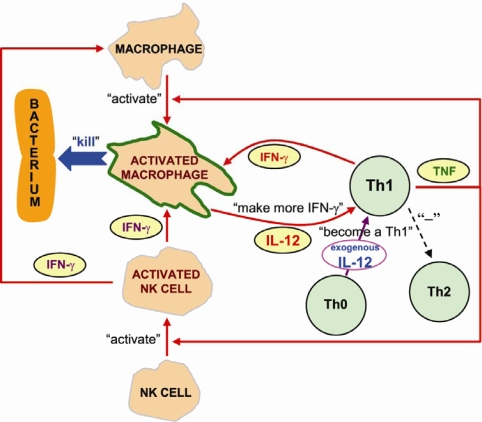
Local IL-12 therapy stimulates Th cells to secrete Th1 cytokines. Exogenous IL-12 application creates an environment rich in IL-12 around the infection site. In such a local environment, newly activated Th cells, responding to the presence of bacteria, exit the blood and are influenced to become Th1 cells and secrete more Th1 cytokines and activate macrophages and NK cells. Activated macrophages will produce more IL-12 and *via* positive feedback, cell-mediated immunity can be promoted to battle bacteria thereby leading to the prevention of infection. Reprinted from Ref. [69].

More recently, studies have shown that IL-12 may play a significant role in preventing osteomyelitis. A decrease in IL-12 production was observed upon pathogen-mediated ligation of MΦ Fcγ or complement receptors [63,64]. This may indicate that there are cases in which regulation of IL-12 production can be affected by pathogens. Perhaps down-regulation of IL-12 has the potential to provide pathogens with a means of suppressing cell-mediated immunity. As a result, it may take a longer time to clear pathogens and lead to a higher risk of developing a bacterial infection. Meanwhile, IL-12 production was shown to decrease following trauma or major burns and results in decreased or delayed cell-mediated immune response and decreased resistance to infection [65–68]. Exogenous application of IL-12, combined with nanotechnology drug delivery systems, may have restored the decreased IL-12 level and recovered the host’s cell-mediated immune response. It has been found to be effective in preventing open fracture-associated osteomyelitis [69,70]. The mechanism of protection is related to the enhancement of Th1 reactivity and activation of MΦs in the early stage of surgery (Figure 5) [69]. The activation of MΦs may lead to enhanced antigen presentation, phagocytosis, Fc receptor expression and nitric oxide and superoxide production [71]. As a result, these changes may substantially increase the ability of MΦs to kill a wide variety of intracellular and extracellular bacteria and to enhance the development of long-term immunity to those pathogens [71].

It is worthy to note that different approaches can be utilized for IL-12 delivery. To name a few, intranasal administration of IL-12 has been used to eradicate osteosarcoma lung metastases [72], and intraperitoneal administration of IL-12 to treat ovarian cancer [73]. Also, IL-12 nanocoatings have been applied for prevention of bone infections [69,70], and IL-12 microspheres for tumor treatments [74]. IL-12 nanocoatings and microspheres may protect IL-12 from losing its bioactivity since IL-12 has a short half-life of 12 hours [75] and IL-12 applied without a delivery vehicle may undergo rapid degradation. Also, studies have shown that treatments with 1000 ng/kg IL-12 in humans [76] and mice [77] were well tolerated.

#### 4.2.2. Tuberculosis

Tuberculosis (TB) is a common and often deadly infectious disease caused by mycobacteria including *Mycobacterium tuberculosis*, *Mycobacterium bovis*, *Mycobacterium africanum*, *Mycobacterium canetti*, and others. The chronic nature of *Mycobacterium tuberculosis* (Mtb) infection has led researchers to investigate the host’s immunity to respond to and control this disease. Previous reports involving TB infection models showed that the development of Th1 cells in response to IL-12 production and subsequent induction of IFN-γ are key players in immunity to TB [78]. Exogenous IL-12 application was found to result in lower bacterial load and increased incidence of survival [79]. DC and Th1 activation and migration to the infected lungs were shown to control bacterial growth *via* IFN-γ–induced activation of phagocytes [80]. This immune response, however, may not be solely mediated by IL-12, as the other IL-12 family members (IL-23, IL-27, and IL-35) may also participate as well [11].

## 5. Conclusions and Outlook

The IL-12 family includes several important immunoregulatory cytokines. Among them, IL-12, IL-23, and IL-27 act on T cells to induce IFN-γ production and promote T cell expansion and differentiation, while IL-35 inhibits T cell proliferation. In the future, it would be of interest to dissect the signaling pathways by which these family members mediate their distinct effects on Th1 and Th2 responses.

IL-12 has multiple biological activities, and it is a key factor that drives Th1 responses and IFN-γ production. Early application or production of IL-12 during infection may activate MΦs and augment a host’s cell-mediated immunity while shaping the ultimate antigen-specific immune responses. As a result, IL-12 may play a key role in protection against bacterial and viral infections, and IL-12 immunotherapy could be of importance in the treatment of diseases where a Th1 response is desirable. Meanwhile, cytokines including IL-12 have a short *in vivo* half-life, and the development of advanced drug delivery systems [81–83] that can protect IL-12 from losing bioactivity would also be of interest.

## Figures and Tables

**Table 1:** Main immunological functions of IL-12.

• Promote naive CD4^+^ T cells to differentiate into Th1
• Enhance the generation and activity of cytotoxic T lymphocytes
• Induce IFN-γ production by (i) NK cell, T cell, DC, and MΦ, (ii) cooperating with B7/CD28 interaction, and (iii) synergizing with IL-18
• Increase MΦ antimicrobial activity
• Prime DC activation to induce more IL-12 production
• Induce functional adhesion molecule expression on Th1 cells and influence T cell trafficking
